# Effectiveness of a Multi-Component Intervention for Overweight and Obese Children (Nereu Program): A Randomized Controlled Trial

**DOI:** 10.1371/journal.pone.0144502

**Published:** 2015-12-14

**Authors:** Noemi Serra-Paya, Assumpta Ensenyat, Iván Castro-Viñuales, Jordi Real, Xènia Sinfreu-Bergués, Amalia Zapata, Jose María Mur, Gisela Galindo-Ortego, Eduard Solé-Mir, Concepció Teixido

**Affiliations:** 1 Asociación Nereu, Alimentación Saludable y Ejercicio Físico, Región Sanitaria de Lleida, Lleida, España; 2 Escuela Superior de Ciencias de la Salud, TecnoCampus Mataró. Universidad Pompeu Fabra, Barcelona, España; 3 Facultad de Enfermería, Universidad de Lleida, Lleida, España; 4 National Institute for Physical Education of Catalonia (INEFC) of Lleida, University of Lleida, Lleida, Spain; 5 Unitat de Suport a la Recerca Lleida, Institut Universitari d’Investigació en Atenció Primària Jordi Gol. USR-IDIAP Jordi Gol, Lleida, Spain; 6 Facultat de Medicina i Ciències de la Salut, Universitat Internacional de Catalunya, Sant Cugat, Barcelona; 7 Nutrition Department, University of Lleida, Lleida, Spain; 8 Centre d'Atenció Primària Primer de Maig. Institut Català de la Salut, Lleida, Spain; 9 Department of Paediatrics Hospital Universitari Arnau de Vilanova, Lleida, Spain; 10 Departament de Medicina, Universitat de Lleida, Lleida, Spain; The Chinese University of Hong Kong, HONG KONG

## Abstract

**Introduction:**

Treatment of childhood obesity is a complex challenge for primary health care professionals.

**Objectives:**

To evaluate the effectiveness of the Nereu Program in improving anthropometric parameters, physical activity and sedentary behaviours, and dietary intake.

**Methods:**

Randomized, controlled, multicentre clinical trial comparing Nereu Program and usual counselling group interventions in primary care settings. The 8-month study recruited 113 children aged 6 to 12 years with overweight/obesity. Before recruitment, eligible participants were randomly allocated to an intensive, family-based multi-component behavioural intervention (Nereu Program group) or usual advice from their paediatrician on healthy eating and physical activity. Anthropometric parameters, objectively measured sedentary and physical activity behaviours, and dietary intake were evaluated pre- and post-intervention.

**Results:**

At the end of the study period, both groups achieved a similar decrease in body mass index (BMIsd) compared to baseline. Nereu Program participants (n = 54) showed greater increases in moderate-intense physical activity (+6.27% vs. -0.61%, p<0.001) and daily fruit servings (+0.62 vs. +0.13, p<0.026), and decreased daily soft drinks consumption (-0.26 vs. -0.02, p<0.047), respectively, compared to the counselling group (n = 59).

**Conclusions:**

At the end of the 8-month intervention, participants in the Nereu Program group showed improvement in physical activity and dietary behaviours, compared to the counselling group.

**Trial Registration:**

ClinicalTrials.gov NCT01878994

## Introduction

Childhood obesity is one of the most important problems in public health. In developed countries, it is considered the most prevalent noncontagious disease and nutritional and metabolic disorder [1]. Treatment and prevention of overweight and obesity in children is of fundamental importance because of the negative physical and psychological impact of these health conditions. Excess body fat in childhood overburdens the musculoskeletal and cardiorespiratory systems, causes psychosocial problems, and has been related to a large number of comorbidities [2–6]. In addition, this excess weight in childhood tends to persist into adulthood [7].

In recent years, interventions have been designed to address this epidemic, as evidenced in numerous meta-analyses and review articles [3,5,8–13]. These studies have shown that the interventions with the greatest impact are multidisciplinary, apply strategies to improve physical activity and dietary habits and in some cases also introduce behavioural or motivational strategies, and include family members, usually the children’s parents [5,10]. Nonetheless, very few interventions are successful and adherence is very low [8,11]. In a review of studies analysing interventions based on treating childhood obesity, Ariza et.al. [8] observed that only 35.1% of interventions were effective in reducing body mass index (BMI) or adiposity. In health care settings, despite being where the problem is diagnosed and where many families are generally predisposed to cooperate in addressing this problem [14–16], the percentages of post-intervention improvement drop to 28.8% with respect to BMI or adiposity, 23.5% for changes in physical activity, and 22% in nutrition habits [8]. Therefore, it is of primary important to create efficient interventions for the treatment of childhood obesity. The primary aim of the present study was to evaluate the effectiveness in the primary health care setting of an intensive, family-based, multi-component, behavioural intervention (Nereu Program) compared to the usual counselling intervention, in terms of anthropometric parameters, physical activity and sedentary behaviours, and dietary intake.

## Materials and Methods

### Design

A randomized, controlled, multicentre clinical trial was designed for children diagnosed with overweight or obesity, randomly allocated to two study arms before recruitment. The Nereu Program (NP) group received an intensive, 8-month, family-based, multi-component behavioural intervention. Assessments were made before (September, 2012) and after the intervention period (June, 2013). The impact of the NP intervention was compared to that observed in the counselling group (CG), an 8-month intervention that provided usual advice on healthy eating and physical activity behaviours.

### Participants

The study included 89 children from Lleida aged 6 to 12 years who were overweight or obese according to the International Obesity Task Force (IOTF) criteria [17], engaged in low levels of activity (less than 2 hours per week of outside of school hours), and for whom complete data were available. Detailed descriptions of the recruitment approaches and ethical aspects of the clinical trial have been previously published [18]. All children were free of medical comorbidities, not using any medications that might affect weight loss or physiological responses to exertion, and had not been enrolled in any other obesity treatment interventions in the previous 6 months. All children and their parents or guardians gave written informed consent. The study protocol was approved by the Clinical Research Ethics Committee (CEIC) of the Primary Care Research Institute IDIAP-Jordi Gol (Registration number: P12/040) and was carried out according to the principles of the Declaration of Helsinki and subsequent revisions [19].

### Randomization

**Fig 1:**
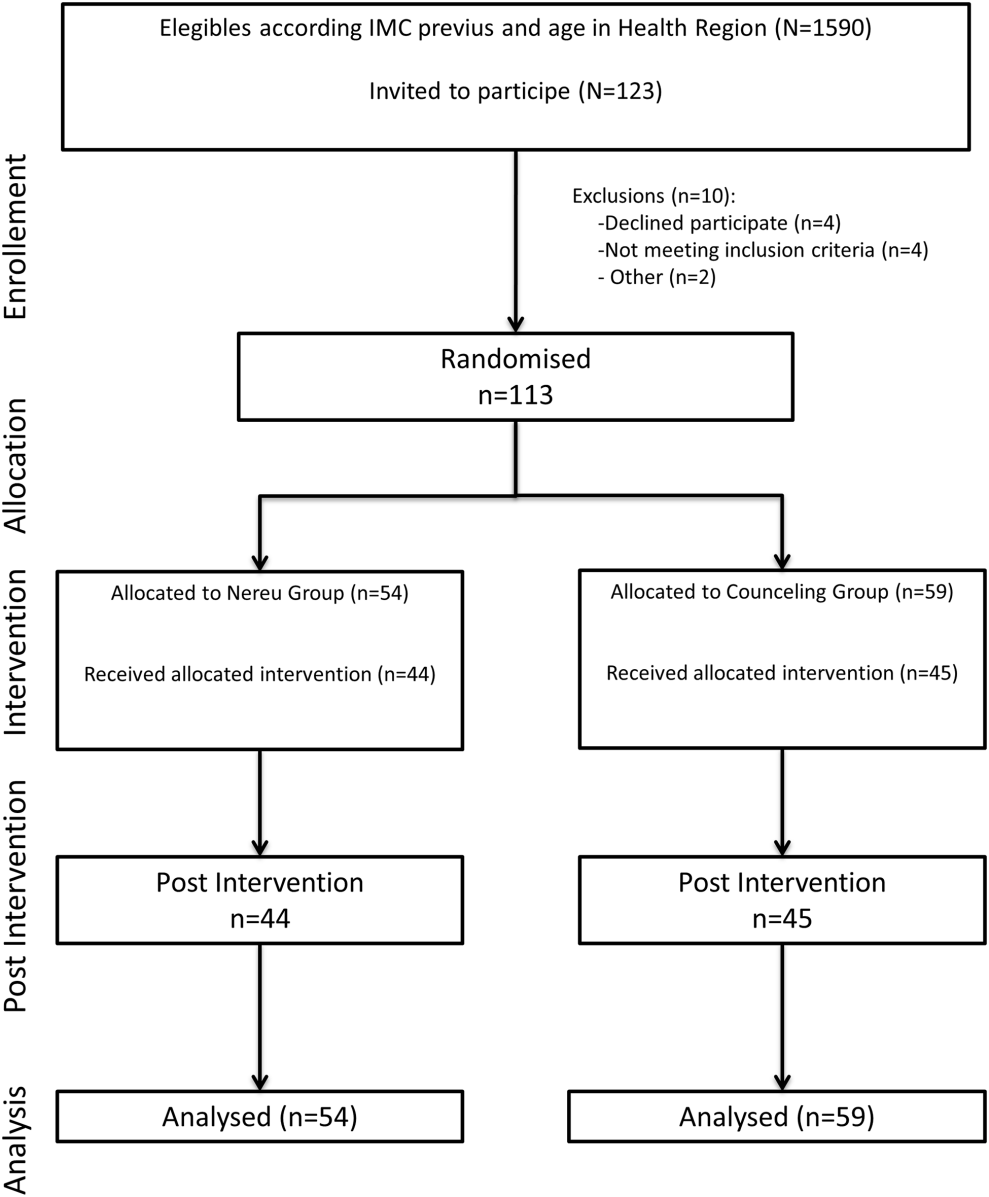
Flow Chart of Study Participants.

Sixteen healthcare paediatrics units (HPU) agreed to participate in this study. Each HPU was responsible for the recruitment and selection of eligible participants. Randomization was centralized at the Primary Care Research Institute (IDIAP) Jordi Gol site in Lleida. Each cooperating healthcare paediatric unit was provided a sort list (using a computer-generated random number) of their eligible patients who met the inclusion criteria (age and BMIsd), according to the data contained in clinical records. These eligible children had been randomly assigned to one of the study arms, stratified by age group in each HPU (Fig 1).

### Sample size

The population of Lleida that met primary inclusion criteria according to our clinical records was 1464 subjects. The aim of the research team was to recruit 50 subjects per study group to achieve at least 100 total participants, estimating a 20% dropout rate. The primary outcome to be achieved by the calculation of sample size was intervention efficacy, defined as a post-intervention reduction in BMIsd scores in a meta-analysis of trials [20]. Accordingly, the sample size was calculated to detect a reduction in BMIsd scores of 1 point (effect size = 0.60), with 80.0% statistical power, and 5% significance level to detect differences between groups with two independent samples.

### Methods

A complete description is presented in the published study protocol [18]; the methods are summarized below.

### Nereu Program

The NP is an intensive, 8-month, family-based multi-component, behavioural intervention in primary care settings, consisting of 4 components: (a) Supervised physical activity sessions for children, (b) Family theoretical and practical sessions for parents, (c) Behaviour strategy sessions for both children and parents, and (d) Weekend activities.

- Supervised physical activity sessions for children. The program offered 90 one-hour sessions (3 per week). The aim was to enhance a physical activity behaviour, enjoy it during physical activity sessions, and learn healthy behavioural habits. The information content of the sessions was the same as their parents’ sessions (b).
- Family theoretical and practical sessions for parents. The 21 theoretical and practical group counselling sessions took place just once a week (60 minutes each) at the same time as the children’s sessions, giving the family the opportunity to exchange experiences and establish shared commitments later at home.
- Behaviour strategy sessions for both (children and parents). Three behaviour strategy sessions were designed to reinforce the acquisition of healthier physical activity and eating habits within the family.
- Weekend activities. Extra family activities (e.g. a ski or water-park party) were organized on 3 weekends, in order to encourage and experience this more active behaviour.

### Counselling group

Eight monthly, 10-minute, structured, family meetings were scheduled with the child’s paediatrics unit. The content of these family sessions was the same as the Nereu Program intervention. Paediatricians were instructed and received all the material for the intervention in advance. The aim was to enhance a physical activity behaviour and learn healthy behavioural habits, in addition to the usual treatment.

### Measurements

All the measurements and questionnaires were administered by the same expert interviewers, who were blinded to the allocated study group in both sessions (baseline and at the end of the intervention).

### Anthropometry

Height, BMI, waist circumference (WC), and waist-to-height ratio (WHtR) were measured following standard procedures. The BMI standard deviation (BMIsd) was determined according to the LMS method for constructing normalized growth standards [21]. Pubertal stage was evaluated by each participant’s paediatrician according to the Tanner classification criteria [22].

### Objective measurement of physical activity and sedentary time

Physical activity and sedentary time were assessed objectively by measurement of free-living movement during awake hours using the ActiGraph GT3X+ accelerometer (ActiGraph LLC, Pensacola, FL, USA) for 8 consecutive days.

Data from accelerometers were analysed using ActiLife 6.0 software (ActiGraph, Pensacola, FL, USA) to determine time spent at different levels of sedentary/activity behaviour. The cut-offs for categorizing the intensity of physical activity were based on trade-offs from validation studies of accelerometer measures [23,24] and defined as follows: sedentary behaviour (SED), less than 100 counts per minute (cpm); light physical activity (LPA), 100 to 2295 cpm; and moderate and vigorous physical activity (MVPA), 2296 to 3999 cpm (3–6 METS).

### Dietary Intake

Dietary intake was assessed using the Food Frequency Questionnaire (FFQ) [25], a quick, easy, and inexpensive method of quantifying food consumption habits. This method has already been used in longitudinal nutritional studies in children [26,27] and to assess eating patterns in children [28]. The questionnaire has been applied in longitudinal studies of different age groups [29].

The present study analysed the primary food groups, based on the FFQ and main nutritional characteristics that were addressed during the intervention: fruits, processed meats, superfluous foods, and soft drinks. The fruits group includes all fruits, which have high levels of antioxidants, fibre, and vitamins, especially A and C complex. The processed meats group includes hamburgers, sausages, pork, and bacon, all of which contain fatty acids (with a high percentage of saturated fats), cholesterol, and very few micronutrients such as vitamins and minerals [30]. The “superfluous” group consists of cookies, industrially produced pastries, dairy-based desserts (e.g., ice cream) and French fries, characterized by a high level of lipid content and/or simple sugars. This high calorie density is unnecessary because it does not contribute major quantities of vitamins and minerals [31]. The final FFQ group, carbonated beverages and industrially produced juice drinks, has high simple sugars content and a notable absence of nutrients [32]. Responses obtained from the FFQ were extrapolated to recommended daily frequencies from the Catalan government’s Public Health Agency [33].

### Data analysis

Intent-to-treat (ITT) analysis was performed for all recruited participants who completed the basal evaluation (n = 113), independently of whether or not they completed the entire protocol and could be included in further analysis (n = 89). The intent-to-treat analysis was carried out as follows: missing values were replaced by the scale scores of the previous assessment (the last observation carried forward [LOCF]) to assure no increase. The initial analysis evaluated the homogeneity of basal characteristics between the study groups using the two-tailed Student *t* test for independent samples for quantitative variables and chi-square for nominal variables (Table 1); quantitative parameters were expressed using the number of valid values (n), the mean and standard deviation (sd) and qualitative parameters using relative frequency (n) and percentages (%).

**Table 1:** Baseline Measurements of the Nereu Program (NP) and Counselling Group (CG).

Basal Parameters		Nereu Program (n = 54)			Counselling (n = 59)	
	n	mean	(sd)	n	mean	sd
**Anthropometric measurements**						
**Height (cm)**	54	143.05	(10.25)	59	142.42	(12.77)
**Weight (kg)**	54	52.54	(13.29)	59	50.83	(12.64)
**BMI (kg/m** ^**2**^ **)**	54	25.22	(3.35)	59	24.65	(3.18)
**BMIsd** ^a^	54	2.47	(0.51)	59	2.42	(0.55)
**WHtR (unit)**	53	0.60	(0.05)	59	0.58	(0.05)
**WC (cm)**	53	85.87	(10.68)	59	83.13	(9.68)
**Active and sedentary behaviour** ^b^						
**SED (% awake time/day)** ^b^	53	35.97	(16.28)	55	34.23	(14.86)
**LPA (% awake time/day)** ^b^	53	50.87	(11.84)	55	51.64	(10.31)
**MVPA (% awake time/day)** ^b^	53	13.17	(6.24)	55	14.13	(6.58)
**Eating habits**						
**Fruits (pieces/day)**	42	1.97	(1.16)	48	2.07	(1.33)
**Processed meats (servings/day)**	42	1.48	(0.86)	48	1.45	(0.90)
**Fish (servings/day)**	42	0.62	(0.39)	48	0.65	(0.49)
**Vegetables (servings/day)**	42	1.93	(1.59)	48	1.67	(1.35)
**Legumes/Pulses (servings/day)**	42	0.24	(0.15)	48	0.21	(0.19)
**Superfluous foods (servings/day)**	42	3.42	(2.65)	48	3.64	(2.75)
**Sugar-sweetened juices and soft drinks (servings/day)**	42	1.12	(1.81)	48	0.69	0.62)

BMI, body mass index (weight (kg)/height (m)^2^); BMIsd, body mass index standard deviation; MVPA, moderate and vigorous physical activities; LPA, light physical activities; SED, sedentary behaviours.

Data shown are mean (SD)

^a^ BMIsd according to LMS method

^b^ % calculated from daily time recorded

To evaluate the impact on quantitative parameters after 8 months of intervention in both groups, the change from basal values was calculated and significant differences between groups were determined using the Student *t* test (Analogous test from interaction of two repeated measures: ANOVA) for independent samples (Tables 2 and 3). Absolute effect was estimated using the difference in means between groups for the observed changes and the 95% confidence interval (95%CI) for normal approximation. Standardized effect size (SES) was computed as the mean difference between the intervention and control group divided by the standard deviation of the control measurement. Interpretation effect sizes: Values 0.2–0.5 represent small changes, 0.5–0.8 moderate changes, and >0.8 large changes. Furthermore, percentage increase over baseline was calculated (Δ%).

**Table 2:** Pre-post Intervention Differences in Main Outcomes between Nereu Program (NP) and Counselling Group (CG).

Outcome	Nereu Program (n = 54)			Counselling (n = 59)			Differences between groups			
	Δmean	(sd)	Δ%	Δmean	(sd)	Δ%	Δmean	95%CI	p-value	SES
**Anthropometric measurements**										
**BMIsd** ^a^	-0.12	(0.22)	-4.94	-0.09	(0.23)	-3.61	-0.03	(-0.12–0.05)	0.415	0.15
**WHtR (unit)**	0.00	(0.03)	-0.77	-0.01	(0.06)	-1.20	0.00	(-0.02–0.02)	0.792	0.04
**WC (cm)**	0.98	(3.89)	1.14	0.80	(8.46)	0.96	0.18	(-2.31–2.67)	0.888	0.02
**Active and sedentary behaviour** ^b^										
**SED (% awake time/day)** ^b^	-2.52	(6.55)	-7.00	0.97	(3.70)	2.85	-3.49	(-5.45–−1.53)	0.001	0.94
**LPA (% awake time/day)** ^b^	-3.76	(5.93)	-7.40	-0.37	(3.16)	-0.73	-3.39	(-5.14–−1.64)	<0.001	1.07
**MVPA(% awake time/day)** ^b^	6.27	(7.78)	47.64	-0.61	(2.76)	-4.31	6.88	(4.74–9.02)	<0.001	2.50
**Eating habits**										
**Fruits (servings/day)**	0.62	(1.36)	31.51	0.13	(0.95)	6.05	0.50	(0.05–0.94)	0.026	0.52
**Processed meats (servings/day)**	-0.79	(0.94)	-53.23	-0.53	(0.79)	-36.68	-0.26	(-0.58–0.07)	0.118	0.32
**Fish (servings/day)**	0.06	(0.30)	9.08	-0.01	(0.42)	-1.62	0.07	(-0.07–0.20)	0.336	0.16
**Vegetables (servings/day)**	0.40	(2.05)	20.86	0.13	(0.78)	7.83	0.27	(-0.32–0.86)	0.346	0.35
**Legumes/Pulses (servings/day)**	0.01	(0.12)	6.10	0.00	(0.13)	-2.18	0.02	(-0.03–0.07)	0.425	0.15
**Superfluous foods (servings/day)**	-0.92	(2.19)	-26.94	-1.00	(2.51)	-27.35	0.07	(-0.81–0.96)	0.868	0.03
**Sugar-sweetened juices/soft drinks (servings/day)**	-0.26	(0.75)	-23.31	-0.02	(0.52)	-3.17	-0.24	(-0.48–0.00)	0.049	0.46

BMIsd, body mass index standard deviation; MVPA, moderate and vigorous physical activities; LPA, light physical activities; SED, sedentary activities.

Data are means (SD), difference, and 95%CI

^a^ BMI (sd) according to LMS method

^b^ % calculated from daily time recorded

sd: Standard deviation

SES: Standardized effect size was computed as the mean difference between intervention and control group divided by the standard deviation of the control measurement.

Interpretation effect sizes: Values 0.2–0.5 represent small changes, 0.5–0.8 moderate changes and >0.8 large changes.

Δ: Increase over baseline

**Table 3:** Differences in Outcomes Between Nereu Program (NP) and Counselling Group (CG) by Sex and Age.

	Nereu Group			Counselling Group			Differences			
By Sex	ΔMean	(sd)	Δ%	ΔMean	(sd)	Δ%	Mean	(95%CI)	p-value	SES
**Active and sedentary behaviours** ^a^ **, Boys**										
SED (% awake time/day)	-3.25	(7.39)	-8.91	-0.02	(3.45)	-0.06	-3.232	(-6.37–−0.10)	0.044	0.94
LPA (% awake time/day)	-4.50	(5.87)	-9.03	0.38	(3.26)	0.75	-4.873	(-7.43–−2.31)	<0.001	1.49
MVPA (% awake time/day)	7.74	(10.02)	56.65	-0.37	(2.90)	-2.45	8.110	(4.04–12.18)	<0.001	2.80
**Active and sedentary behaviours** ^a^ **, Girls**										
SED (% awake time/day)	-1.78	(5.63)	-5.04	2.24	(3.69)	6.70	-4.020	(-6.65–−1.39)	0.003	1.09
LPA (% awake time/day)	-3.03	(6.01)	-5.83	-1.33	(2.80)	-2.48	-1.703	(-4.30–0.89)	0.191	0.61
MVPA (% awake time/day)	4.81	(4.31)	37.96	-0.91	(2.59)	-7.10	5.720	(3.76–7.68)	<0.001	2.21
**By Age Group**										
**Active and sedentary behaviours** ^**a**^ **, Pre-puberty**										
SED (% awake time/day)	-1.08	(4.14)	-4.56	0.73	(3.92)	2.93	-1.81	(-4.52–0.89)	0.183	0.46
LPA (% awake time/day)	-5.78	(5.00)	-9.65	-0.08	(3.41)	-0.13	-5.70	(-8.51–−2.89)	<0.001	1.67
MVPA (% awake time/day)	6.86	(5.55)	41.59	-0.65	(2.72)	-3.75	7.51	(4.36–10.66)	<0.001	2.76
**Active and sedentary behaviours** ^**a**^ **, Puberty**										
SED (% awake time/day)	-3.12	(7.29)	-7.57	1.11	(3.62)	2.86	-4.23	(-6.88–−1.58)	0.002	1.17
LPA (% awake time/day)	-2.92	(6.15)	-6.21	-0.54	(3.04)	-1.11	-2.38	(-4.61–−0.15)	0.036	0.78
MVPA (% awake time/day)	6.03	(8.60)	51.35	-0.58	(2.81)	-4.66	6.61	(3.66–9.57)	<0.001	2.35

BMIsd, body mass index standard deviation; SED, sedentary activities; LPA, light physical activities; MVPA, moderate and vigorous physical activities.

Data are means (SD)

^a^ % calculated from daily time recorded

Data are means (SD), difference, and 95%CI

SES: Standardized effect size was computed as the mean difference between intervention and control group divided by the standard deviation of the control measurement.

Interpretation effect sizes: Values 0.2–0.5 represent small changes, 0.5–0.8 moderate changes and >0.8 large changes

Δ: Increase over baseline

Linear correlation between the number of sessions attended (program adherence) and changes produced in the NP group compared to CG participants was assessed for statistical significance using the Pearson correlation coefficient. Analysis of subgroups by sex and age was used to determine sensitivity and evaluate possible interactions between the effects analysed.

All analyses were carried out using the SPSS statistical packages v. 18 (SPSS Corp). The level of significance was set at *p*=.05.

## Results

The present study included 113 boys and girls and their respective families (Fig 1), randomized into two study groups, 54 in the NP group and 59 in the CG. No significant basal differences were observed between the two groups in age (mean 10.1±1.98 vs. 9.73±1.97years), pubertal stage (Tanner 1 83.3% vs. 82.1%), or sex (50% boys vs. 44.1% girls), respectively (Table 1). Longitudinal analysis of the mean changes observed in the major parameters after completing the intervention is shown in Table 2. Post-intervention change in BMIsd was -0.12 in the NP group and -0.09 in the CG, with no significant difference between the two groups.

At the end of the intervention, the NP group reduced both SED and LPA (-2.52% and -3.76%, respectively) and increased the time dedicated to MVPA (+6.27%), significant differences (p<0.001) compared to the CG, which increased SED and decreased both LPA and MVPA time (+0.97%, -0.37%, and -0.61%, respectively). Notably, the 7.78% increase in MVPA observed in the NP participants represents more than 1 hour/day, bringing the total to 2.85 hours/day of MVPA (Table 2).

With respect to eating habits, after the 8-month intervention the NP participants increased their fruit consumption compared to the CG (+0.62 vs. +0.13 piece/day, p = 0.026), and decreased their intake of processed meats (-0.79 vs. -0.53 servings/day, p = 0.118) and sugar-sweetened juices and soft drinks (-0.26 vs. -0.02 servings/day, p = 0.047), respectively. Both groups reduced their intake of superfluous foods (-0.92 vs. -1.00 servings/day, respectively).

At the beginning of the study, an analysis stratified by sex and age found no differences between any subgroups (Table 3). At the end of the interventions, the differences observed between NP and CG participants in changes in the time dedicated to MVPA and SED were also reflected in all sub-groups except the pre-pubertal age group, where there was no difference between study arms in SED habits (p = 0.183). The post-intervention increase in MVPA was 7.7% for the subgroup of NP boys, and 4.8% for NP girls. Stratified by age, MVPA increased 6.9% for the pre-pubertal NP participants and 6% for the pubertal NP subgroup.

Adherence to the intervention program was similar in both groups, with mean attendance of 51% (46 of 90 sessions) for the NP group and 47.8% for CG participants (n = 4 of 8 sessions). In the NP group, there was a direct correlation between adherence (number of sessions attended) and reduction in anthropometric parameters (BMIsd, r = 0.547, p<0.001; WHtR, r = 0.373, p = 0.014; and WC, r = 0.359, p = 0.018). In all subgroups by sex and age, adherence to program attendance was similar in both study groups.

## Discussion

The aim of this study was to evaluate the effectiveness of the Nereu Program as an intensive, family-based, multi-component behavioural intervention in achieving recommended anthropometric parameters and physical activity, sedentary behaviour and dietary intake goals, compared to a standard counselling intervention. After the 8-month intervention, the NP group had improved activity habits (SED, LPA, and MVPA), especially notable in their increase in MVPA to more than 2.5 hours daily. Improvements were also achieved in eating habits, with more than half an additional serving of fruit per day and a one-third decrease in daily servings of sugar-sweetened juices and soft drinks. In both groups, improved anthropometric parameters were observed; however, participants in the NP group who attended the most sessions achieved the greatest reduction in BMIsd, WC, and WHtR.

In the present study, there was high adherence to attending intervention sessions in both groups. This is a very encouraging result, given that the NP intervention consisted of 90 sessions and both interventions covered an 8-month period. Low adherence is typically one of the major limitations of health care interventions [8,34]. In addition, greater adherence in terms of number of sessions attended was correlated with greater anthropometric improvement in the NP group.

In a meta-analysis by Waters et al. of 37 interventions [5], the mean change in BMIsd after the intervention was -0.15 (p = 0.01). The Oslo Adiposity Intervention Study showed a decline of 0.13 in BMIsd [35], similar to that observed in both the NP and CG participants in the present study. These are encouraging results because this represents a beneficial change in paediatric health. Small reductions in BMIsd have been related with metabolic improvements and lower insulin resistance [35,36].

The increase in time spent daily in MVPA and the decrease in SED time after participation in the NP intervention are very encouraging results. These improvements represent an increase in MVPA to more than 2.5 hours daily, surpassing the recommended minimum of 1 hour/day [24]. After the intervention, NP participants spent 3.7 hours/day in SED behaviours, close to the 3.61 hours/day observed in children aged 9–10 years participating in the European Youth Heart Study (EYHS) and less than the 5.59 hours/day recorded by adolescents (14–15 years old) [37], reflecting observation made by other researchers that as children grow up, they become more sedentary and spend less time in MVPA [37–39].

Physical activity is a crucial component of treatment for childhood obesity, due both to the benefits of regular exercise for weight reduction and to the general health improvements that have been reported [40,41]. Comparing the present results with previous studies, 45% of interventions designed to prevent childhood obesity obtain increases in physical activity; in contrast, the rate of effectiveness decreases to 23.5% for treatment interventions [8]. Improvements in active and sedentary habits in the NP group occurred homogeneously across subgroups of sex and age. This is an important outcome because it is known that sports participation decreases with age and that boys tend to be more physically active than girls [38,39]. These findings suggest that the NP intervention was a beneficial program for both boys and girls of all ages (6 to 12 years).

The highlight among the improvements in eating habits was the increased consumption of fruits in the NP group, reaching nearly 3 daily servings; this nutritional intake is considered ideal [42,43]. According to Lock et al. [44], 31% of cardiovascular disease cases are due to low consumption of fruits and vegetables; therefore, healthy eating habits have a significant long-term impact on cardiovascular health [44]. The improved fruit intake in the NP group exceeded the level recorded for Spain in the European Health Survey, in which about 38% of the total population reported eating two or more pieces of fruit daily [43] and 14% of children ate the recommended 3 daily [42,43]. We must also emphasize the large decline in superfluous foods consumed, such as cakes or industrially produced pastries. This is due in great part to the replacement of these products with fruit for dessert. The results also showed that NP participants reduced the consumption of processed meats with high lipid content. This is very encouraging and highly beneficial because eating these products increases cholesterol and changes the population lipid profile, causing a greater long-term risk of arteriosclerosis, angina pectoris, or cerebrovascular accidents [45]. These products are also laden with sodium, a cause of hypertension and increased blood pressure [46]. Another favourable outcome, considering current trends, was the decreased consumption of sugar-sweetened juices and soft drinks. Consumption of these beverages is increasingly prevalent in our setting, representing about 8% of daily calorie intake in the general population’s eating habits [47].In addition, the habit of drinking canned beverages such as colas has been associated with increased bone demineralization because of the high phosphorous content, which can lead to osteoporosis in the long term. Both soft drinks and industrially produced juices contain excessive amounts of simple sugars, which can lead to type II diabetes, overweight, obesity, and other cardiovascular diseases [48].

In the short-term results, the NP intervention proved useful in changing physical activity and sedentary behaviours and improving eating habits, but no significant differences in anthropometric parameters were observed between the study arms. Therefore, we must wait to see if these newly acquired habits are maintained and have long-term anthropometric effects. It will be important to evaluate the mid- and long-term status of these changes.

## Limitations

The present study has several limitations that must be considered. Despite high program adherence, the rate of losses and missing values affected the effect size, depending on the parameter, which limited the statistical power to detect differences between groups in the changes observed. Nonetheless, the losses were balanced between the study arms, eliminating the threat of differential bias. The FFQ has certain limitations, even though it was completed jointly by parents and their children to improve accuracy. There are, of course, occasions when parents are unaware of what their children eat outside of the home, such as school lunches or meals eaten at a grandparent’s house. Even with the child’s participation, it is sometimes difficult to reflect food intake with precision. It is important to point out, however, that the FFQ was administered by expert interviewers using food models to improve the reliability of the results and also that this limitation would be equally present in both study arms.

## Conclusions

After completing the NP intervention, an intensive, family-based, multi-component, behavioural intervention delivered within a universal primary health care program, significant improvements were observed in activity and eating habits of participating children, compared to the CG intervention. There was an increase in time dedicated to MVPA and a reduction in SED time. Fruit consumption increased more than half a piece per day and daily servings of sugar-sweetened juices and soft drinks decreased by nearly one third of a daily serving. Despite these encouraging short-term results, it remains to be seen if the observed improvements persist at mid-term and long-term follow-up.

## Supporting Information

S1 CONSORT Checklist CONSORT Checklist. (DOC)

S1 Protocol Protocol. (PDF)

## Abbreviations

BMI: Body Mass Index
BMIsd: Body Mass Index Standard Deviation
CG: Counselling Group
MVPA: Moderate and Vigorous Physical Activities
NP: Nereu Program
SED: Sedentary Activities

## References

[1] World Health Organization website. Factsheet. Available: http://www.who.int/mediacentre/factsheets/fs311/es/. 2014.

[2] Baker J, Olsen L, Sorensen T. Childhood Body-Mass Index and the Risk of Coronary Heart Disease in Adulthood. *N Engl J Med*. 2007;357(23):2329–2337. 18057335 10.1056/NEJMoa072515 PMC3062903

[3] Bibbins-Domingo K, Coxson K, Pletcher MK, Lightwood J, Goldman L. Adolescent Overweight and Future Adult Coronary Heart Disease. *N Engl J Med*. 2007;357:2371–2379. 10.1056/NEJMc073592 18057339

[4] Dehghan M, Akhtar-danesh N, Merchant AT. Childhood obesity, prevalence and prevention. *Nutr J*. 2005;4(24):1–8. 10.1186/1475-2891-4-24 16138930 PMC1208949

[5] Waters E, De Silva-Sanigorski A, Hall BJ, Brown T, Campbell KJ, Gao Y, et al Interventions for preventing obesity in children (Review). Cochrane Database Sytemtic Rev. 2011;(12). 10.1002/14651858.CD001871.pub3 22161367

[6] Weiss R, Dziura J, Burgert TS, Tamborlane WV, Taksali SE, Yeckel CW, et al Obesity and the Metabolic Syndrome in Children and Adolescents. *N Engl J Med*. 2004;350:2362–2374. 15175438 10.1056/NEJMoa031049

[7] Whitaker RC, Wright JA, Pepe MS, Seidel KD, Dietz WH. Predicting obesity in young adulthood from childhood and parental obesity. *N Engl J Med*. 1997;337(13):869–873. 9302300 10.1056/NEJM199709253371301

[8] Ariza C, Ortega-Rodríguez E, Sánchez-Martínez F, Valmayor S, Juárez O, Pasarín MI. La prevención de la obesidad infantil desde una perspectiva comunitaria. *Atención Primaria*. 2015;47(4):246–255. 10.1016/j.aprim.2014.11.006 25835135 PMC6985614

[9] Whitlock EP, O’Connor EA, Williams SB, Beil TL, Lutz LK. Effectiveness of Primary Care Interventions for Weight Management in Children and Adolescents: An Updated, Targeted Systematic Review for the USPSTF. Evid Syntesis. 2010;76: 1–106. 20722175

[10] Spruijt-Metz D. Etiology, treatment, and prevention of obesity in childhood and adolescence: A decade in review. *J Res Adolesc*. 2011;21(1):129–152. 10.1111/j.1532-7795.2010.00719.x Etiology. 21625328 PMC3102537

[11] Oude-Luttikhuis H, Baur L, Jansen H, Shrewsbury VA, O'Malley C, Stolk RP, et al Interventions for treating obesity in children (Review). *Cochrane Database Sytematic Rev*. 2009;3(1):1–57. 10.1002/14651858.CD001872.pub2 19160202

[12] Lavelle HV, Mackay DF, Pell JP. Systematic review and meta-analysis of school-based interventions to reduce body mass index. J Public Health (Bangkok). 2012;34(3):360–369. 10.1093/pubmed/fdr116 22267291

[13] Sargent GM, Pilotto LS, Baur LA. Components of primary care interventions to treat childhood overweight and obesity: a systematic review of effect. Obes Rev. 2011;12(5):219–235. 10.1111/j.1467-789X.2010.00777.x 20630025

[14] McCallum Z, Wake M, Gerner B, Harris C, Gibbons K, Gunn J. Can Australian general practitioners tackle childhood overweight/obesity? Methods and processes from the LEAP (Live, Eat and Play) randomized controlled trial. *J Paediatr Child Health*. 2005;41:488–494. 16150065 10.1111/j.1440-1754.2005.00689.x

[15] Albright CL, Cohen S, Gibbons L, Miller S, Marcus B, Sallis J, et al Incorporating physical activity advice into primary care: physician-delivered advice within the activity counseling trial. *Am J Prev Med*. 2000;18(3):225–234. 10.1016/S0749-3797(99)00155-5 10722989

[16] Ortega R, Jiménez C, Córdoba R, Muñoz J, García ML, Vilaseca J. The effect of office-based physician’s advice on adolescent exercise behavior. Prev Med (Baltim). 2004;38(2):219–226. 10.1016/j.ypmed.2003.09.042 14715215

[17] Cole TJ, Bellizzi MC, Flegal KM, Dietz WH. Obesity Worldwide: International Survey. *Bmj*. 2000;320:1–6. 10.1136/bmj.320.7244.1240 10797032 PMC27365

[18] Serra-Paya N, Ensenyat A, Real J, Castro-Viñuales I, Zapata A, Galindo G, et al Evaluation of a family intervention programme for the treatment of overweight and obese children (Nereu Programme): a randomized clinical trial study protocol. BMC Public Health. 2013;13:1000 10.1186/1471-2458-13-1000 24153001 PMC4015510

[19] Declaration of Helsinki (2013). Ethical principles for medical research involving human subjects. *WMA*. http://www.wma.net/en/ 30publications/10policies/b3/index.html.

[20] Wilfley DE, Tibbs TL, Van Buren DJ, Reach KP, Walker MS, Epstein LH: Lifestyle interventions in the treatment of childhood overweight: a meta-analytic review of randomized controlled trials. Health Psychol 2007, 26(5):521–532. 17845100 10.1037/0278-6133.26.5.521 PMC2040042

[21] Cole JL. The LMS method for constructing normalized growth standards. *Eur J Clin Nutr*. 1990;44(1):45–60. 2354692

[22] Lupash E. Acsm’s guidelines for exercise testing and prescription. American College of Sports Medicine. Acsm's guidelines for exercise testing and prescription. Am Coll Sport Med. 2009. 10.1249/JSR.0b013e31829a68cf 23851406

[23] Aznar Laín S, Webster T. Actividad física y salud en la infancia y la adolescencia *Guía para todas las Pers que Particip en su Educ Minist Educ*. 2006.

[24] World Health Organization (WHO). Recomendaciones mundiales sobre actividad física para la salud. Geneva WHO Libr Cat Data 2010.

[25] Martin-Moreno JM, Boyle P, Gorgojo L, Maisonneuve P, Fernandez-Rodriguez JC, Salvini S, et al Development and validation of a food frequency questionnaire in Spain. *Int J Epidemiol*. 1993;22(3):512–519. 8359969 10.1093/ije/22.3.512

[26] Field AE, Peterson KE, Gortmaker SL, Cheung L, Rockett H, Fox MK, et al Reproducibility and validity of a food frequency questionnaire among fourth to seventh grade inner-city school children: implications of age and day-to-day variation in dietary intake. *Public Heal Nutr*. 1999;2:293–300. 10.1017/s1368980099000397 10512564

[27] Rockett HR, Wolf AM, Colditz GA. Development and reproducibility of a food frequency questionnaire to assess diets of older children and adolescents. *J Am Diet Assoc*. 1995;95(3):336–340. 7860946 10.1016/S0002-8223(95)00086-0

[28] Sabaté J. Estimación de la ingesta dietética: métodos y desafíos. Med Clin (Barc). 1993;100:591–596. 8497151

[29] Aguirre-Jaime A, Cabrera de León A, Domínguez SC, Borges C, Carrillo L, Gavilán JC, et al Validation of a Food Intake Frequency Questionnaire Adapted for the Study and Monitoring of the Adult Population of the Canary Islands, Spain nos factore. Rev Esp Salud Publica. 2008;82(5):509–518. 19039504 10.1590/s1135-57272008000500006

[30] Burriel F, Urrea R, García C, Tobarra M, Meseguer MJG. Hábitos alimentarios y evaluación nutricional en una población universitaria. *Nutr Hosp*. 2013;28(2):438–446. 10.3305/nh.2013.28.2.6303 23822696

[31] Heitor SFD, Rodrigues LR, Santiago LB. Introdução de alimentos supérfluos no primeiro ano de vida eas repercussões nutricionais; Introduction of junk food in the childÆs first year and its nutritional outcome; Introducción de alimentos superfluos en el primer año de vida y las repercusiones nu. Ciênc Cuid saúde. 2011;10(3):430–436.

[32] Villaresa JM, Segoviab MG. Supervisión de la alimentación en la población infantil y juvenil. *Rev Pediatría Atención Primaria*. 2008;10(39):165–168.

[33] Agència Salut Pública (2006). La Agencia de Salud Pública de Cataluña. Documento de bases para su creación. *General Catalunya Dep Salut* 2006. Available: http://www.gencat.es:8000/salut/depsalut/pdf/docbase2007.pdf.

[34] Barja Y, Nuñez N, Velandia A, Urrejola N, Hodgson B, Isabel M. Adherencia y efectividad a mediano plazo del tratamiento de la obesidad infantil: compliance and outcome over medium term. Rev Chil pediatría. 2005;76(2):151–158.

[35] Kolsgaard ML, Joner G, Brunborg C, Anderssen SA, Tonstad S, Andersen LF. Reduction in BMI z-score and improvement in cardiometabolic risk factors in obese children and adolescents. The Oslo Adiposity Intervention Study—a hospital/public health nurse combined treatment. *BMC Pediatr*. 2011;11(1):47 10.1186/1471-2431-11-47 21619652 PMC3121603

[36] Feliu Rovira A, París Miró N, Zaragoza-Jordana M, Ferré N, Chiné M, Sabench F, et al Eficacia clínica y metabólica de una nueva terapia motivacional (OBEMAT) para el tratamiento de la obesidad en la adolescencia. An Pediatría. 2012;78(3). 10.1016/j.anpedi.2012.06.006 22832041

[37] Van-Sluijs EM, Page A, Ommundsen Y, Griffin SJ. Behavioural and social correlates of sedentary time in young people. *Br J Sports Med*. 2010;44(10):747–755. 10.1136/bjsm.2008.049783 18812418

[38] Troiano RP, Berrigan D, Dodd KW, Mâsse LC, Tilert T, McDowell M. Physical Activity in the United States Measured by Accelerometer. Med Sci Sports Exerc. 2008;40(1):181–189. 18091006 10.1249/mss.0b013e31815a51b3

[39] Ruiz JR, Ortega FB, Martínez-Gómez D, Labayen I, Moreno LA, De Bourdeaudhuij I, et al Objectively Measured Physical Activity and Sedentary Time in European Adolescents The HELENA Study. *Am J Epidemiol*. 2011;174(2):173–184. 10.1093/aje/kwr068 21467152

[40] Norman AC, Drinkard B, McDuffie JR, Ghorbani S, Yanoff SB, Yanovski JA. Influence of Excess Adiposity on Exercise Fitness and Performance in Overweight Children and Adolescents. *Pediatrics*. 2005;115(6):e690–e696. 15930197 10.1542/peds.2004-1543 PMC1350764

[41] Peyrot N, Thivel D, Isacco L, Morin JB, Duche P, Belli A. Do mechanical gait parameters explain the higher metabolic cost of walking in obese adolescents? *Appl Physiol*. 2009;106:1763–1770. 10.1152/japplphysiol.91240.2008 19246657

[42] Majem LS, Barba LR, Bartrina JA, Rodrigo CP, Santana PS, Quintana LP. Obesidad infantil y juvenil en España. Resultados del Estudio enKid (1998–2000). Med Clin (Barc). 2003;121(19):725–732. 14678693 10.1016/s0025-7753(03)74077-9

[43] Encuesta Europea de Salud en España. Available: http://www.msssi.gob.es/estadEstudios/estadisticas/EncuestaEuropea/Principales_Resultados_Informe.pdf. 2009.

[44] Lock K, Pomerleau J, Causer L, Altmann DR, McKee M. The global burden of disease attributable to low consumption of fruit and vegetables: implications for the global strategy on diet. *Bull World Health Organ*. 2005;83(2):100–108. 15744402 PMC2623811

[45] Van’t Veer P, Jansen MC, Klerk M, Kok FJ. Fruits and vegetables in the prevention of cancer and cardiovascular disease. *Public Health Nutr*. 2000;3(01):103–107. 10.1017/S1368980000000136 10786730

[46] Pacheco Pérez WA, Arias Muñoz CE, Restrepo Molina DA. Effect of Reduction of Sodium Chloride on the Quality Characteristics of a Sausage Type Selected. 2012;65(2):6779–6787.

[47] Rubio AM, PrevInfad MG, Infancia PAPPS. Supervisión de la alimentación en la población infantil y juvenil. Rev pediatría atención primaria. 2008;10(37):99–133.

[48] Rubio AM, Domínguez JD. Recomendaciones dietéticas en la infancia y adolescencia. La pirámide nutricional como instrumento didáctico. *Rev Pediatría Atención Primaria*. 2008;10(2):139–153.

